# A Novel Arc Fault Detector for Early Detection of Electrical Fires

**DOI:** 10.3390/s16040500

**Published:** 2016-04-09

**Authors:** Kai Yang, Rencheng Zhang, Jianhong Yang, Canhua Liu, Shouhong Chen, Fujiang Zhang

**Affiliations:** 1 College of Mechanical Engineering and Automation, Huaqiao University, Xiamen 361021, China; yangknh@126.com (K.Y.); yjhong@hqu.edu.cn (J.Y.); xsjtan@sina.com (C.L.); shouhong55@163.com (S.C.); zfj@fjut.edu.cn (F.Z.); 2 School of Mechanical and Automotive Engineering, Fujian University of Technology, Fuzhou 350118, China

**Keywords:** arc fault detector (AFD), electrical fire, high-frequency energy, current variation, weighted least squares support vector machine, cross talk

## Abstract

Arc faults can produce very high temperatures and can easily ignite combustible materials; thus, they represent one of the most important causes of electrical fires. The application of arc fault detection, as an emerging early fire detection technology, is required by the National Electrical Code to reduce the occurrence of electrical fires. However, the concealment, randomness and diversity of arc faults make them difficult to detect. To improve the accuracy of arc fault detection, a novel arc fault detector (AFD) is developed in this study. First, an experimental arc fault platform is built to study electrical fires. A high-frequency transducer and a current transducer are used to measure typical load signals of arc faults and normal states. After the common features of these signals are studied, high-frequency energy and current variations are extracted as an input eigenvector for use by an arc fault detection algorithm. Then, the detection algorithm based on a weighted least squares support vector machine is designed and successfully applied in a microprocessor. Finally, an AFD is developed. The test results show that the AFD can detect arc faults in a timely manner and interrupt the circuit power supply before electrical fires can occur. The AFD is not influenced by cross talk or transient processes, and the detection accuracy is very high. Hence, the AFD can be installed in low-voltage circuits to monitor circuit states in real-time to facilitate the early detection of electrical fires.

## 1. Introduction

As reported by the United States Fire Administration (USFA), there were an estimated 372,900 residential building fires in the United States each year from 2011 to 2013, causing an estimated 13,125 injuries, 2530 deaths and $7 billion in property damage [1]. Fires represent a serious threat to humans. Electrical fires are one of the most frequently occurring types of fires, representing on average up to 30.2% of all fires and exceeding 50% of all large fires [2,3]. Statistical data from fire administrations show that electrical fires are usually caused by arc faults, over currents, short circuits, leakages [1,2,3,4,5,6], *etc*. After the USFA studied the sources of heat in residential building electrical fires, they stated that arc fault accounted for 82% of the electrical fires [6]. Specifically, arc faults represent one of the most important causes of electrical fires [1,5,6]. Traditional fire detection technologies usually monitor smoke, gas, temperature, *etc.* Recently, several fire feature signals could be detected by multisensors [7]. The multi-wavelength LiDAR technique was applied to study the smoke of a fire [8]. A laser spectroscopic carbon monoxide sensor was used to detect fires [9]. Some wireless sensors and intelligent technologies were introduced for fire identification [10,11,12]. These technologies typically detect fires after they have occurred. Various advanced early detection methods may be able to effectively reduce fire losses and the number of false alarms. Early detection technologies can protect electrical equipment and humans before fires occur. Consequently, the development of advanced early detection technologies for reducing the occurrence of electrical fires caused by arc faults represents an urgent task.

**Figure 1:**
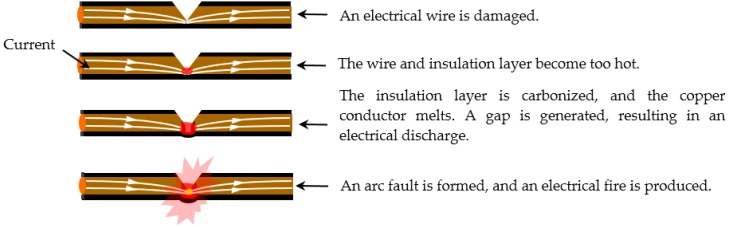
An electrical fire resulting from an arc fault.

What is an arc fault? According to the Underwriters Laboratories (UL) Standard UL 1699, arcing is defined as a luminous electrical discharge across an insulating medium, and the partial volatilization of the electrodes is usually accompanied by the arcing process. An arc fault is defined as an unintended arcing situation in a circuit [13]. However, visible sparks, or arcs, naturally generated in relays and brush motors are different from arc faults. Such sparks are arcs generated under normal operation and are considered to be non-hazardous in terms of fire detection. Arc faults are generated in circuits for many reasons. Typical causes include electrical cords or wires having loose connections; worn insulation on electrical cords or wires resulting from corrosion, age, bending stress or heat; and damaged or misapplied electrical equipment. Figure 1 shows a process of an electrical fire caused by an arc fault. The insulating material layer of the damaged electrical wire is carbonized, and sporadic arcs are subsequently generated. Over time, the carbonized paths are further expanded, and severe arcs are formed. These arcs can release a tremendous amount of energy and can reach temperatures of up to 13,000 K [14]. If combustible materials are nearby, a fire will easily be produced [14,15].

Arc faults are categorized into three types: series arc faults, parallel arc faults and ground arc faults. As a result of the existence of fault arc impedance, the currents of series arc faults are usually lower than the rated currents of loads, and the currents of parallel and ground arc faults are usually lower than those of short circuits. The arc fault currents are not sufficient to trigger traditional protection devices such as overcurrent protectors and ground fault circuit interrupters. Therefore, these protection devices cannot be used to protect circuits from arc faults [16]. Moreover, arc faults are often latent, intermittent and transient; thus, one should monitor circuit states in real-time to detect such faults to prevent electrical fires. Hence, an arc fault detector (AFD) represents an urgent need in electrical fire detection.

Arc fault circuit interrupters (AFCIs) represent an important AFD for preventing fires. The president of the National Association of State Fire Marshals (NASFM), John C. Bean, stated that “NASFM strongly supports the broad adoption of AFCI technology through national, state, and local building codes. AFCIs are the most welcome addition to fire prevention in decades. AFCIs promise to save hundreds of lives every year” [17] (page number of quote). Moreover, the USFA stated that “The Consumer Product Safety Commission (CPSC) has identified arc fault circuit interrupter (AFCI) technology as an effective means of preventing fires caused by electrical wiring faults in homes” [17] (page number of quote). UL has released the standard UL 1699: UL standard for safety arc-fault circuit-interrupters [13], and the International Electrotechnical Commission (IEC) has released the standard IEC 62606: general requirements for arc fault detection devices [18]. Furthermore, the National Electrical Code (NEC) requires the installation of AFCIs.

Because the detection method is the main component of an AFCI, many researchers have studied arc fault detection technologies. Some researchers have developed mathematical arc fault models such as an instantaneous arc model for a resistive-inductive system [19,20], a simplified arc fault model for analyzing and determining basic parameters [21,22,23], and an arc fault meta model of conductance [24]. Many studies have shown that arc faults exhibit some abnormal behaviors in terms of arc radiation, arc sounds, arc light, arc temperatures, arc currents and arc voltages in circuits [25,26,27,28,29]. When arc faults occur in circuits, the radiated electromagnetic signals increase in intensity and can be collected using a loop or stick antenna [25]; sounds and light from fault arcs appear and significantly increase in intensity [26,27]; and arc temperatures quickly increase to up to 13,000 K, with a large amount of heat being instantaneously released to the surrounding area [14]. Fault arc voltages in transmission lines can be estimated through the least error squares method and can be used to identify arc faults [28,29]. However, mathematical arc fault models are usually used in theoretical analysis and simulations. Arc radiation, arc sounds, arc light, arc temperatures and arc voltages are often characterized by randomness because the times and locations of arc faults are always random. Therefore, arc fault detection approaches based on such behaviors have certain limitations when used in practice.

Line currents, which are easily measured in circuits, have been used to study arc fault features in many studies. Many high-frequency components have been found in the currents of arc faults, the frequency bands of which are usually wide [30,31]. The spectral energy of currents has been calculated to discriminate arc faults from load states [32]. Some arc fault detection algorithms have been implemented in microcontrollers [33,34], and various patents for arc fault detection devices have been approved [35,36,37,38]. However, the accuracies of many detection devices have not been high [39]. Because arc fault features are always hidden in the line currents, many advanced methods have been used to extract them. A Kalman filtering algorithm, artificial neural networks and fuzzy logic algorithms have been separately used to classify arc faults and normal states [40,41,42]. The harmonic components of arc fault currents have been analyzed [43,44]. The reconstructed information entropy of each current frequency band has been calculated to obtain the feature frequency bands of arc faults [45]. Arc fault currents have been decomposed based on the discrete wavelet transform (DWT), where the wavelet transform coefficients were used as fault features [46]. In one study, currents were first de-noised by DWT to improve the signal-to-noise ratio, and the current eigenvectors were then trained through wavelet networks to detect arc faults [47]. The Gabor Transform (GT) has been used to obtain an optimal feature extraction; the extracted GT coefficients of currents were used to identify arc faults [48].

However, arc fault currents are always strongly affected by different types of loads. Fault currents produced by certain home appliances do not change noticeably, and arc fault features are usually easily masked by load currents and background noise. It is difficult to find a general fault feature that performs well for all loads. Moreover, the residential electrical standard for single-phase alternating current (AC) is 120 V (60 Hz) in the United States, whereas it is 220–240 V (50–60 Hz) in other countries including China, Germany, Switzerland and Korea. Higher voltages more readily produce electrical fires caused by arc faults [49,50,51,52]. For example, the probability of fire ignition due to arc faults increases from 3.5% for 120 V to 83% for 240 V, and their nominal current levels are both 15 A [49]. Compared to 120 V, the higher voltages from 220 to 240 V are more likely to break down gaps and lead to more arcs [50,51]. Arc currents are usually continuous in higher voltage systems, but they are sometimes intermittent in 120 V systems [49,50]. Thus, higher voltage systems generate larger arc energy and thus provide better conditions for the ignition of electrical fires [50,51].

Furthermore, some commercial AFCIs often fail to trip when necessary or trip when they should not; the accuracy of arc fault detection was only approximately 50% in a previously published research report [39]. Consequently, it remains difficult to accurately detect all arc faults in circuits, and certain detection methods continue to require refinement, especially for 220–240 V operation.

In this study, to increase the accuracy of arc fault detection, a novel AFD will be developed for the early detection of electrical fires. First, many currents and high-frequency signals from low-voltage circuits will be acquired to study the general features of arc faults. Then, common features will be extracted and used to identify arc faults using an advanced identification method. Compared to general identification algorithms, such as neural network algorithms, support vector machine (SVM)-based algorithms can obtain global optimums and have good generalization ability, especially when addressing high dimensions or small samples [53,54,55]. A weighted least squares support vector machine (WLS-SVM) algorithm will be introduced for arc fault identification in this study. Finally, the arc fault detection algorithm will be applied to hardware toward developing an AFD.

The remainder of the paper is organized as follows. In Section 2, an experimental platform for electrical fire research is built, and a substantial amount of experimental data is collected. In Section 3, different types of load signals are analyzed to find the common features of arc faults, and high-frequency energy and current variations are extracted as the eigenvector for arc fault detection. Section 4 presents the arc fault detection algorithm based on WLS-SVM. In Section 5, the final design of the AFD for use in the early detection of electrical fires is introduced, and the performance of the AFD is tested. Finally, Section 6 concludes this paper.

## 2. Experiments

**Figure 2:**
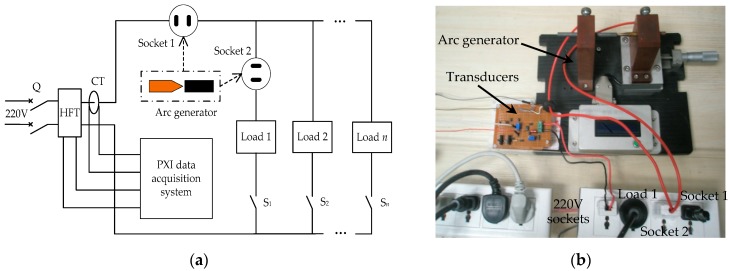

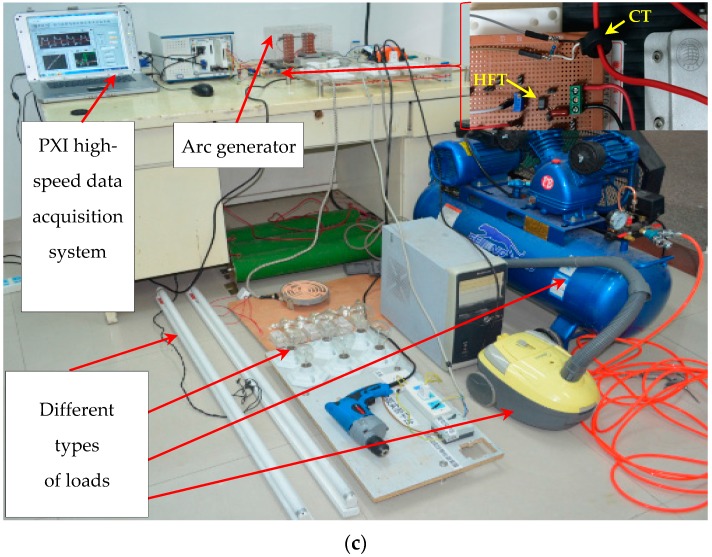
Experimental platform for electrical fire research: (**a**) schematic; (**b**,**c**) actual platform.

The experimental arc fault platform for electrical fire research mainly consists of electrical loads, an arc generator, transducers, and a data acquisition system. Figure 2 shows a schematic and the actual experimental platform. The platform is built based on the relevant standards for arc faults in low-voltage circuits. The standards include IEC 62606: 2013, UL 1699 and GB/T 14287.4-2014 [13,18,56]. Typical electrical loads for the experiments are listed in Table 1.

**Figure 3:**
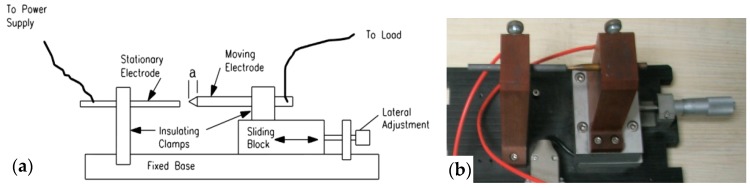
The arc generator: (**a**) structure; (**b**) the actual device.

**Figure 4:**
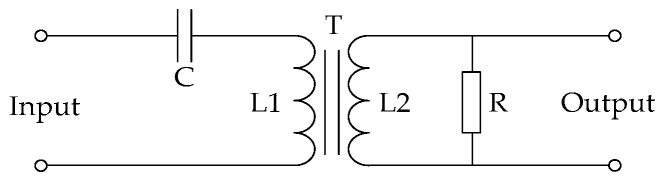
The HFT schematic.

The arc generator includes a moving electrode and a stationary electrode, as shown in Figure 3. One electrode is a carbon-graphite rod 6.4 mm in diameter, and the other electrode is a copper rod. The arc generator is used to generate arc faults in circuits. The transducers consist of a high-frequency transducer (HFT) and a current transducer (CT), as shown in Figure 2c. A schematic of the HFT is shown in Figure 4. The input and output terminals of the transducer are connected to the power supply terminals of the loads and data acquisition system, respectively. A transducer CT210 is selected as the CT. A PXI-5124 digitizer fixed in PXIe-1071 is used for high-speed data acquisition, and the sample rate is set as 125 MS/s. As shown in Figure 4, the high-frequency signals in the power supply circuit are coupled by a capacitor C and a transformer T. A high-pass filter composed of a capacitor C and the primary coil L1 of T is used. The power frequency signals at 50 Hz will be first filtered by the high pass filter. According to the principle of electromagnetic induction, many high-frequency current signals will be generated in the secondary coil L2. Then, the high-frequency voltage signals will be generated in a resistor R. Finally, they will be collected by the PXI-5124 digitizer. The transformer T is used as an isolated unit between the strong electricity signal and the weak electricity signal. Both arc faults and normal states of typical loads can be realized using the experimental platform. Large quantities of high-frequency signals and currents in different load states are collected to study the common features of arc faults in the next section.

## 3. Analysis of Experimental Results

### 3.1. High-Frequency Signal Analysis

#### 3.1.1. High-Frequency Signals and Power Spectra

**Figure 5:**
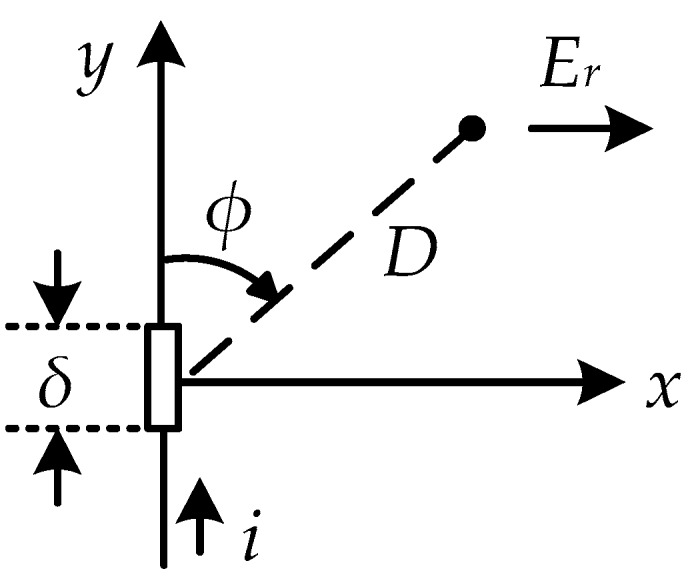
The electromagnetic field along the *x*-axis.

High-frequency signals were found to be a reflection of the dynamic arcing process in many arc fault experiments. Upon the initial arcing, many air molecules between electrodes begin to be ionized; subsequently, the plasma motion quickly intensifies. Because electrode gaps, oxide layers, surface states, adsorbed gases and dielectric materials are usually heterogeneous, this dynamic motion is always uncertain. According to electromagnetic theory, many high-frequency signals can be easily generated in circuits. These signals will spread through conductors or radiate into the air. An electromagnetic field $E_{r}$ is generated by the current *i*, as shown in Figure 5. The value of $E_{r}$ oriented along the *x*-axis can be expressed as:

(1) $$E_{r}\left(D,t\right)\approx \frac{\sin\phi }{4\pi \varepsilon_{0}Dc^{2}}\int_{0}^{\delta }\frac{di}{dt}dx$$

where *D* is the distance from the component, *t* is time, ϕ is the angle with the *y*-axis, $\varepsilon_{0}$ is the vacuum permittivity, *c* is the speed of light, and δ is the length of the current component [57]. For a spot arc, the length δ is negligible; therefore, Equation (1) can be simplified as:

(2) $$E_{a}\left(t\right)\approx \gamma \frac{di_{a}}{dt}=\gamma {\dot{i}}_{a}$$

where γ is a constant.

The power spectrum of the arc fault can be expressed as:

(3) $$P_{a}\left(\omega \right)={\left|\int_{0}^{\infty }E_{a}\left(t\right)e^{-j\omega t}dt\right|}^{2}=\frac{\gamma^{2}{\left[{\dot{i}}_{a}\left(0\right)\right]}^{2}\omega^{2}}{{\left(\mu^{2}-\omega^{2}\right)}^{2}+4\varphi_{}^{2}\omega^{2}}$$

where $R_{a}$ is the arc resistance, $L_{a}$ is the arc inductance, $C_{a}$ is the arc capacitance, $\mu =1/\sqrt{L_{a}^{}C_{a}^{}}$ is the natural frequency of the arc fault, and $\varphi =R_{a}^{}/2L_{a}^{}$ is the arc inductive damping rate [58]. Considering that the fault arc inductance can be ignored, the power spectrum of the fault arc in the circuit can be approximated as:

(4) $$P_{a}\left(\omega \right)\approx \frac{\gamma^{2}{\left[{\dot{i}}_{a}\left(0\right)\right]}^{2}\mu^{2}\omega^{2}R_{a}^{2}C_{a}^{2}}{1+R_{a}^{2}C_{a}^{2}\omega^{2}}$$

**Figure 6:**
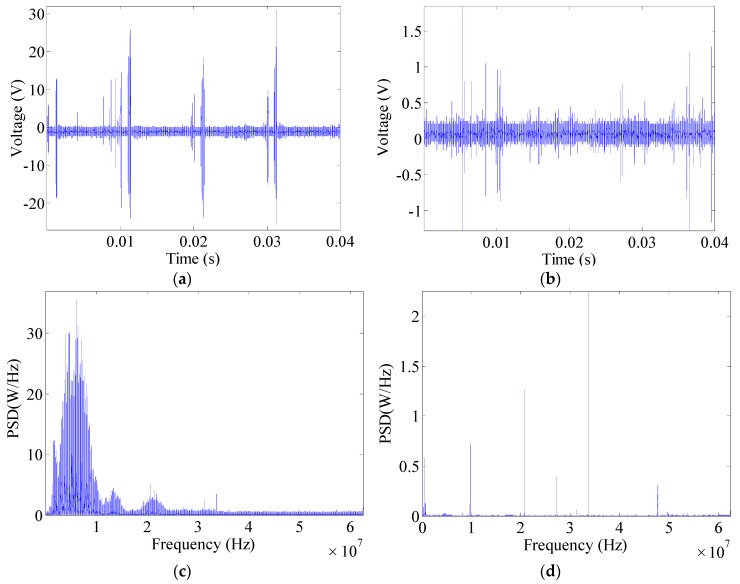
Signal analysis of an electrical drill: (**a**) high-frequency signals of arc faults; (**b**) high-frequency signals under normal operation; (**c**) PSD of arc fault signals; (**d**) PSD under normal operation.

An electrical drill’s high-frequency signals and corresponding power spectral density (PSD) are shown in Figure 6. The high-frequency signals of arc faults are densely distributed and exhibit large amplitudes. Various frequency bands with large amplitudes can also be found in the power spectrum. However, when an electrical drill is operated in the normal state, random interference pulses are generated in circuits. The pulses are caused by a brush motor in the electrical drill. Electrical discharges are usually accompanied by friction between the brush and rotor. Electrical sparks are easily observed from the electrical drill’s windows. These sparks are load arcs and are not hazardous. Interference generated by these sparks should be overcome in arc fault detection. Therefore, the varied regularities of spectra over time will help facilitate further analysis.

#### 3.1.2. Short-Time Fourier Transform (STFT) of High-Frequency Signals

**Figure 7:**
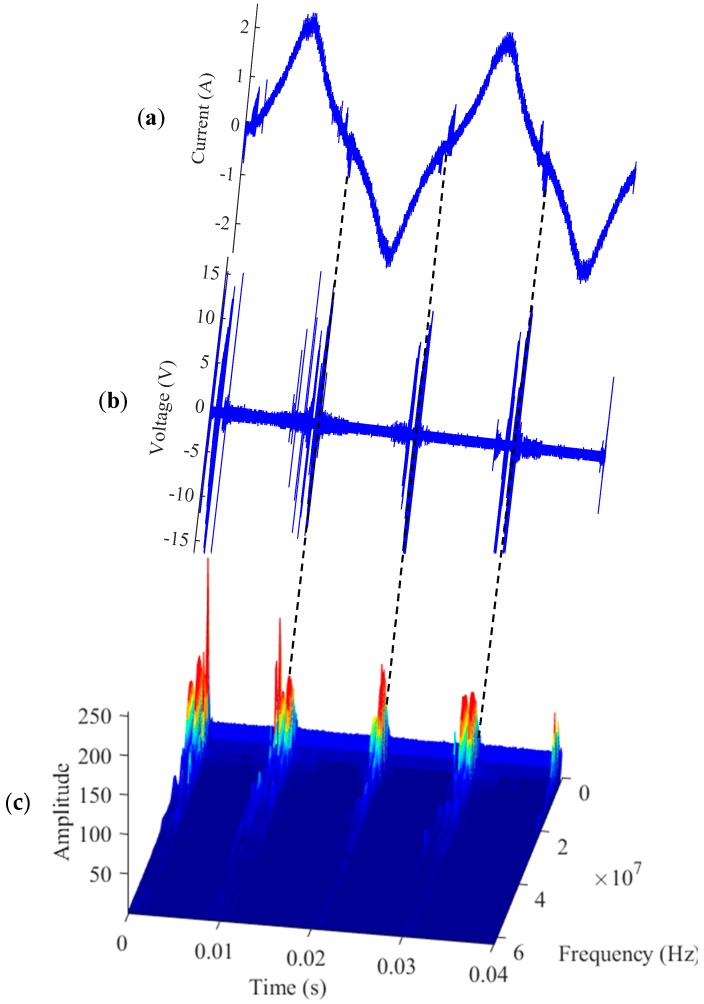
Signals’ STFT for the electrical drill. (**a**) line current; (**b**) high-frequency signals; (**c**) signals’ STFT of arc faults.

Because arc faults are non-stationary, their frequency spectra and power spectra are usually time variant. To further research the features of arc faults in different time and frequency bands, STFT is introduced. The arc fault signals’ STFT for the electrical drill is shown in Figure 7. The high-frequency signals of the arc faults can be found in both the line current and phase voltage, and they are generated periodically following the current cycles, as shown in Figure 7a,b. They clearly change in a wide frequency band, especially under 30 MHz, as shown in Figure 7c.

**Figure 8:**
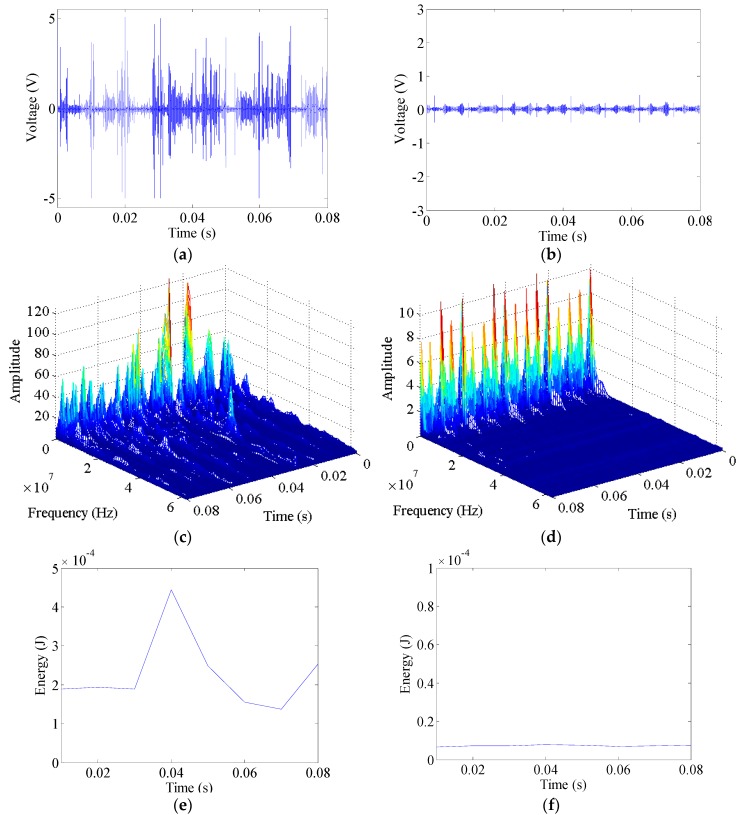
Signal analysis of a vacuum cleaner: (**a**) high-frequency signals of arc faults; (**b**) high-frequency signals under normal operation; (**c**) signals’ STFT of arc fault signals; (**d**) signals’ STFT under normal operation; (**e**) high-frequency energy of arc faults; (**f**) high-frequency energy under normal operation.

**Figure 9:**
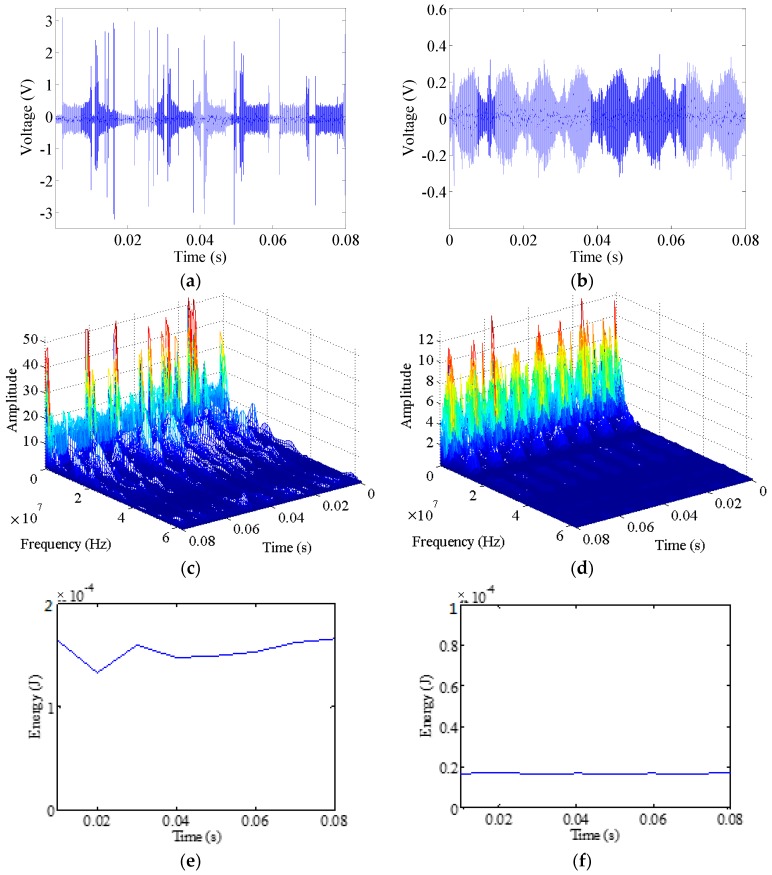
Signal analysis of halogen lamps: (**a**) high-frequency signals of arc faults; (**b**) high-frequency signals under normal operation; (**c**) signals’ STFT of arc fault signals; (**d**) signals’ STFT under normal operation; (**e**) high-frequency energy of arc faults; (**f**) high-frequency energy under normal operation.

To find the common features of arc faults, many high-frequency signals produced by different typical loads are collected, as shown in Figure 8 and Figure 9. Substantial power grid noise is generated by power lines, and some short pulses are initially generated when the vacuum cleaner operates in the normal state, as shown in Figure 8b. The power grid noise mainly originates from power line carrier communication, switching power supplies, load faults, *etc.* According to the provisions from the State Grid Corporation of China, the power line carrier frequency band is 10–500 kHz [59]. Some frequencies of other strong noise sources in the power grid can be up to 1.5 MHz [59,60]. According to the signals’ STFT under normal operation, the large amplitude noise contains frequencies that are also mainly under 1.5 MHz, as shown in Figure 8d. The switch transformers in the halogen lamps often alternate between on and off states; they are controlled based on the pulse width modulation. Therefore, many high-frequency signals are also periodically generated when operating in the normal states of the halogen lamps, some frequencies of which are up to hundreds of kHz [61]. The normal signal frequencies with large amplitudes are mostly less than 1.5 MHz, as shown in Figure 9d.

Broadband frequency components from arc faults can be clearly found in Figure 7c, Figure 8c and Figure 9c. The amplitudes decrease with increasing frequency. The frequency components are not the same at different times. They are also not the same for different types of loads. Noticeable spectrum signatures appear near the zero-crossing regions of arc fault currents, and currents may disappear in a short period of time, as shown in Figure 7a. The zero-crossing regions are also reflected in the figures showing the high-frequency signals and their spectra, as shown in Figure 7b,c, Figure 8a,c and Figure 9a,c. Near the zero-crossing regions, arcs may extinguish themselves. However, they will re-ignite if there are sufficient voltages to reestablish the arcs between the electrodes. As a result of the abrupt changes of the arc currents, many high-frequency signals with large amplitudes are rapidly generated. They can be collected by an HFT, as shown in Figure 7b, Figure 8a and Figure 9a. In the practical engineering, according to the propagation characteristics of electromagnetic interference, conduction propagation is the main propagation mode of electromagnetic interference below 30 MHz [62]. Moreover, according to the signals’ STFT, the high-frequency signals of arc faults under typical loads concentrating in the 1.5–30 MHz frequency band increase more noticeably than those under normal operation, as shown in Figure 7c, Figure 8c,d and Figure 9c,d; moreover, the signals may have greater energy. Therefore, 1.5–30 MHz can be regarded as a feature frequency band.

#### 3.1.3. The Periodic Energy of High-Frequency Signals

The original signals from the HFT are filtered to obtain the high-frequency signals in the 1.5–30 MHz frequency band. The periodic energy $E_{hf}(k)$ of these high-frequency signals can be calculated as:

(5) $$E_{hf}(k)=\int_{(k-1)T_{\mathrm{AC}}}^{kT_{\mathrm{AC}}}s^{2}(t)dt\;(k=1,2,\cdots ,K)$$

where $T_{\mathrm{AC}}$ = 10 ms is half the AC current period for a 50 Hz power grid, also being half an fault arc period, and *s*(*t*) represents the high-frequency signals.

**Figure 10:**
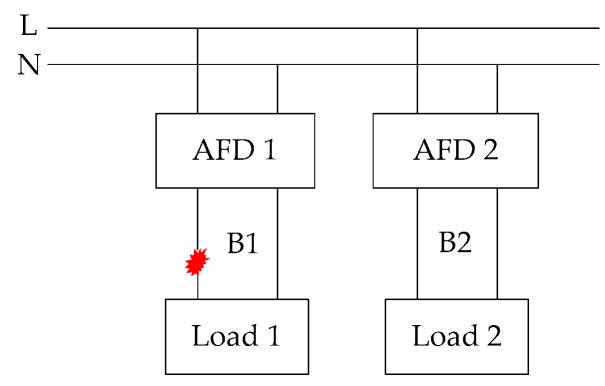
Cross talk.

The vacuum cleaner’s high-frequency energy $E_{hf}(k)$ is calculated as shown in Figure 8e,f, and the halogen lamps’ $E_{hf}(k)$ is calculated as shown in Figure 9e,f. Based on many calculations of $E_{hf}(k)$, the $E_{hf}(k)$ of arc faults is larger than that under normal operating conditions for a load. Nevertheless, the $E_{hf}(k)$ of different loads often varies. It is difficult to select a reasonable constant threshold to discriminate arc faults from the load states for all loads. Furthermore, high-frequency signals are easily influenced by cross talk, as shown in Figure 10. The high-frequency signals in branch B1 can be detected by the HFT in AFD 2, which is in an adjacent circuit. Hence, this will lead to false alarms.

The high-frequency signals can propagate via radio frequency and conduction. Cross talk is formed by electromagnetic coupling. It can be divided into capacitive coupling cross talk and inductive cross talk. Due to the capacitive coupling, the induced current is

(6) $$i_{M1}=C_{M1}\frac{du_{N1}}{dt}$$

where $C_{M1}$ is the coupling capacitance between wires M1 and N1, and $u_{N1}$ is the voltage of the wire N1. If $u_{N1}$ changes quickly, $i_{M1}$ will become large. Thus, the high-frequency signals can generate a large $i_{M1}$. Due to the inductive coupling, the induced voltage is

(7) $$u_{M1}=L_{M1}\frac{di_{N1}}{dt}$$

where $L_{M1}$ is the coupling inductance between wires M1 and N1, and $i_{N1}$ is the current of the wire N1. Similarly, the high-frequency signals can generate a large $u_{M1}$ [62]. Fortunately, the voltage $u_{M1}$ induced by power frequency currents at 50 Hz is small and is easily masked in the power frequency voltages. In other words, power frequency currents at 50 Hz are not easily influenced by cross talk. To overcome the influence of cross talk, the features of currents will be extracted and added for analysis in the next section.

### 3.2. Current Analysis

#### 3.2.1. Typical Load Currents

**Figure 11:**
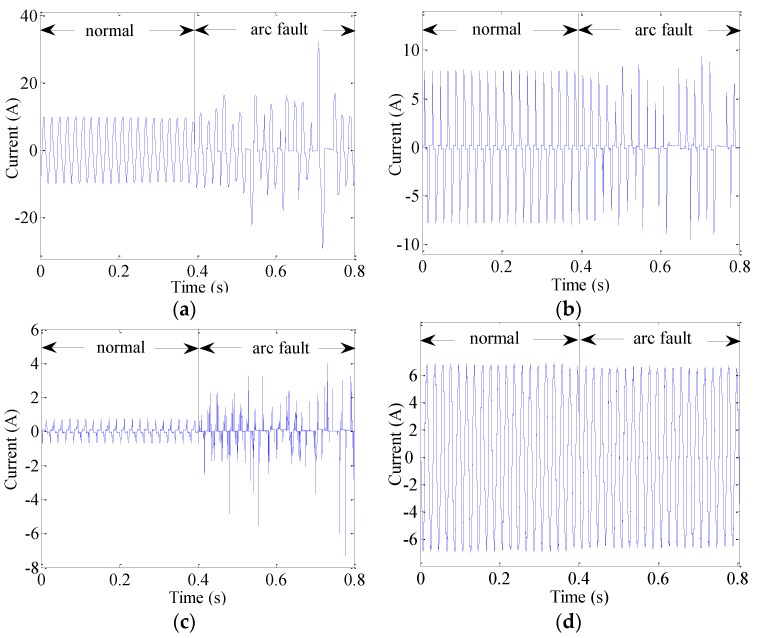
Currents: (**a**) an air conditioner; (**b**) dimming lamps; (**c**) a computer; (**d**) an electric stove.

**Figure 12:**
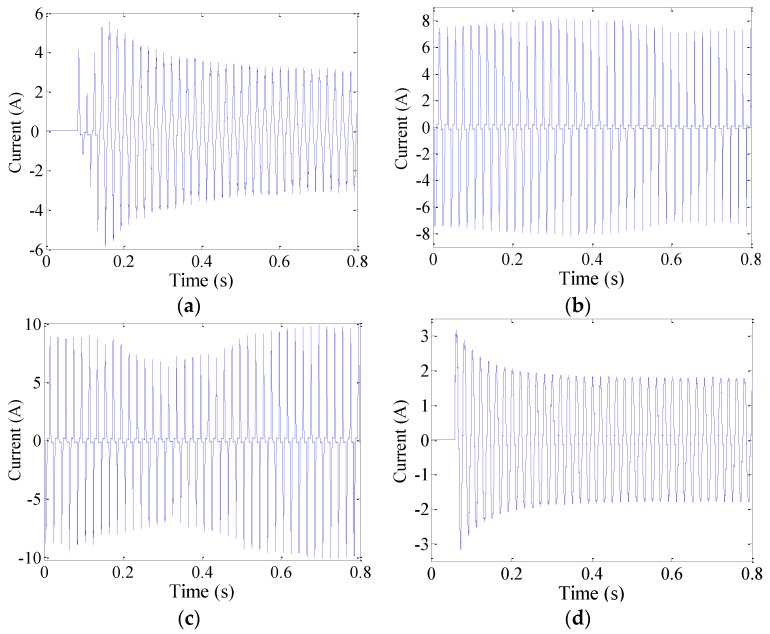
Currents of transient processes: (**a**) starting an electrical drill; (**b**) dimming lamps; (**c**) regulating the speed of a vacuum cleaner; (**d**) plugging in a halogen lamp.

Line currents of various typical loads exhibit various characteristics. When loads operate in stable states, as in the case of “normal” conditions, as shown in Figure 11, currents in circuits remain relatively stable and exhibit good symmetrical characteristics and certain periodicities. When loads can be characterized as transient processes, such as starting loads, dimming lamps, regulating speeds and plugging in or unplugging, currents rapidly change monotonically at first but then return to stable states, as shown in Figure 12. Nevertheless, currents will become random or noticeably distorted, their periodicities will be lost, and their amplitudes will become erratic if there are arc faults in circuits, such as under “arc fault” conditions, as shown in Figure 11. Moreover, current amplitudes may become zero at times. This is because the inductive and capacitive reactances of fault arcs are usually unstable, and they may change sporadically as a result of external conditions. The entire varied regularities of currents from normal conditions to arc faults under different typical loads can be observed in Figure 11. Because current features can be used to overcome the influence of cross talk, current variations should be quantitatively described to accurately extract arc fault features.

#### 3.2.2. Current Feature Extraction

**Figure 13:**
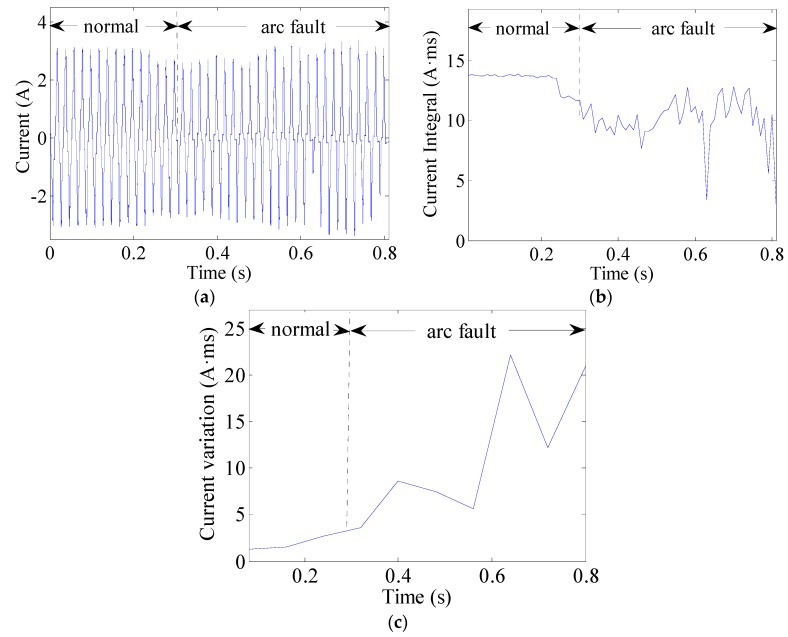
Arc fault current analysis of the electrical drill: (**a**) current waves; (**b**) current periodic integrals; (**c**) current variations.

**Figure 14:**
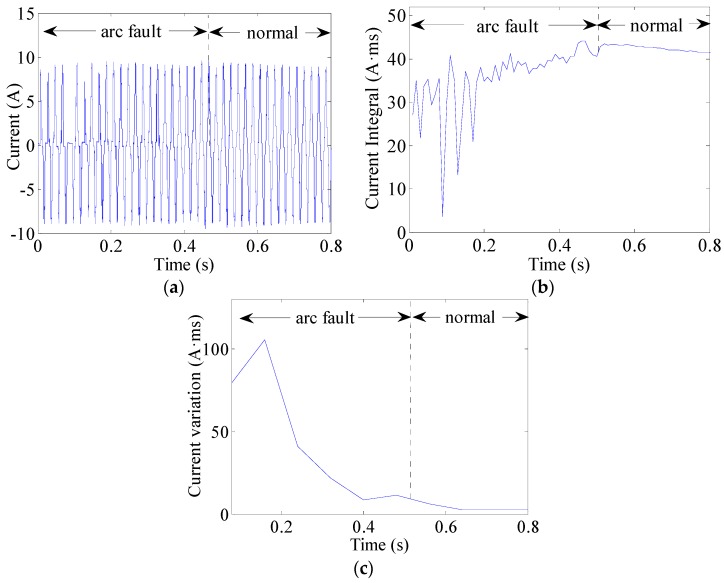
Arc fault current analysis of the vacuum cleaner: (**a**) current waves; (**b**) current periodic integrals; (**c**) current variations.

**Figure 15:**
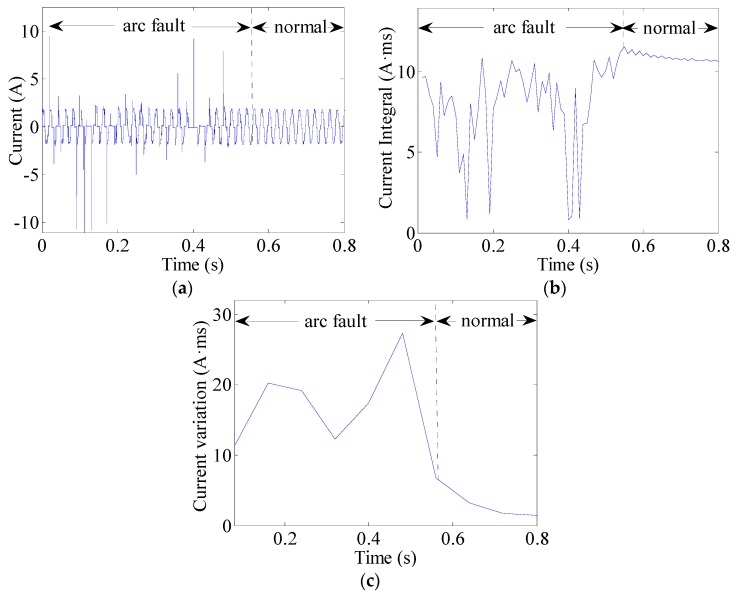
Arc fault current analysis of the halogen lamps: (**a**) current waves; (**b**) current periodic integrals; (**c**) current variations.

**Figure 16:**
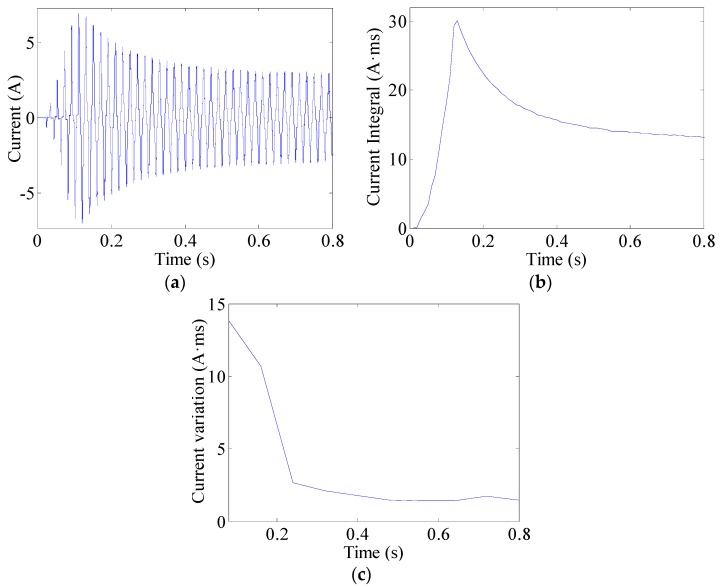
Transient process analysis of the electrical drill: (**a**) current waves; (**b**) current periodic integrals; (**c**) current variations.

**Figure 17:**
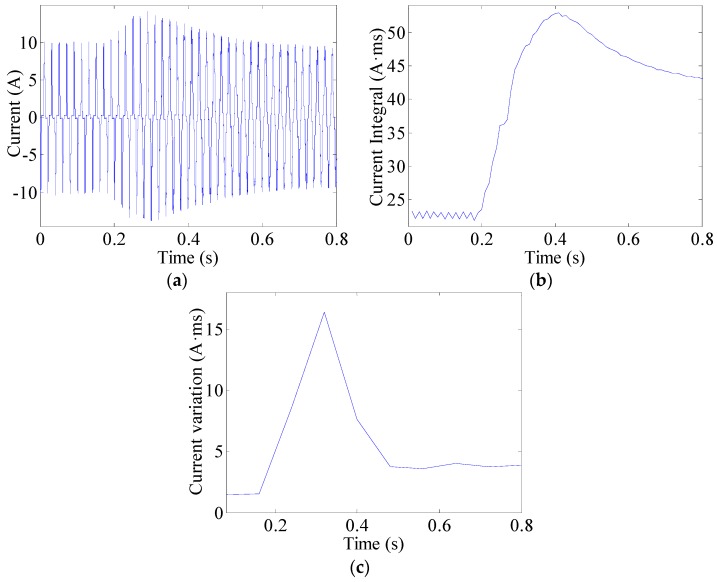
Transient process analysis of the vacuum cleaner: (**a**) current waves; (**b**) current periodic integrals; (**c**) current variations.

The current integral can be used to observe changes in currents. This can be expressed as

(8) $$J(k)=\int_{(k-1)T_{\mathrm{AC}}}^{kT_{\mathrm{AC}}}\left|i(t)\right|dt\;(k=1,2,\cdots ,K)$$

where $T_{\mathrm{AC}}$ = 10 ms is half the AC current period and *i*(*t*) represents the line current. An arc fault is clearly a stochastic process. The line currents of arc faults are often seriously distorted or change in uncertain manners, as shown in Figure 13a, Figure 14a and Figure 15a. Current periodic integrals of arc faults often fluctuate and become erratic, as shown in Figure 13b, Figure 14b and Figure 15b. Nevertheless, the changes in current periodic integrals can also be observed when loads operate under normal conditions, especially during transient processes. The electrical drill current during startup and its periodic integrals are shown in Figure 16a,b, respectively. The vacuum cleaner current when regulating speed and its periodic integrals are shown in Figure 17a,b, respectively. The current periodic integrals also change during these transient processes; they change in terms of regularity and remain monotonic over a certain period of time. Hence, there are some limitations in identifying arc faults from transient processes only via current periodic integrals.

To avoid incorrect determinations during transient processes, the current variations in several current periods are introduced. According to the geometric meaning of definite integrals, the definite integral can be used to represent the total area of a graph. This area contains positive and negative areas such as the current wave area over a current period. The current period is 20 ms. Load current waves of arc faults are often asymmetrical, whereas those of normal states are symmetrical. Therefore, the definite integrals in AC current periods can be used to represent current variations. The current variations can be calculated as the summation of several current periodic integrals over a certain period of time expressed as:

(9) $$J_{sum}(k)=\sum_{k=1}^{K}\left|\int_{(k-1)T_{\mathrm{AC}}}^{(k+1)T_{\mathrm{AC}}}i(t)dt\right|\;(k=1,2,\cdots ,K)$$

where $T_{\mathrm{AC}}$ = 10 ms is half the AC current period and K = 8; therefore, the total time is 80 ms. The current variations of the electrical drill, vacuum cleaner and halogen lamps are shown in Figure 13c, Figure 14c and Figure 15c, respectively. From the analysis of many current variations, we find that the current variation thresholds between arc faults and normal states are unequal under different loads. Taking start-up states and arc faults as examples, some current variations in the electrical drill startup state are similar to those of the arc faults of halogen lamps, as shown in Figure 15c and Figure 16c. This may hence lead to false identification.

In conclusion, the high-frequency energy of arc faults clearly increases, and the current periodic integrals of arc faults randomly change. Nevertheless, the high-frequency signals are easily influenced by cross talk, and the use of only current variations may result in false identification under different loads. To accurately detect arc faults, several features, including the high-frequency energy and current variations, should be used simultaneously. Furthermore, the development of an intelligent detection algorithm also represents a very important and urgent need. The detection algorithm will be introduced and detailed in the next section.

## 4. Arc Fault Detection Algorithm

Compared to general identification algorithms, such as neural network algorithms, SVM-based algorithms can avoid falling into local minima, over learning, the dimensional disaster [63,64], *etc*. Such algorithms can obtain a global optimum and have good generalization ability, especially when addressing high dimensions or small samples [53,54,55]. Based on the traditional SVM, Suykens and Vandewalle presented the LS-SVM algorithm, which can ensure satisfactory identification accuracy while reducing the computational complexity [53]. To more accurately detect arc faults, the high-frequency energy and current variations are selected as the eigenvector. They can be calculated using Equations (5) and (9), respectively. Based on the input eigenvector ***y***, the WLS-SVM algorithm will be applied to arc fault detection. The WLS-SVM algorithm has emerged as an improved algorithm for enhancing the sparseness and robustness of LS-SVM [65].

A nonlinear function *f*(***y***) is used to map the eigenvector ***y*** from the observation space to a higher dimensional feature space. The optimal identification between arc faults and normal states can be obtained in the higher dimensional feature space. The constrained optimization problem can be described as [53,65]:

(10) $$\left\{ \begin{array}{l} \underset{U,\zeta_{n},q}{\min}W\left(U,\zeta_{n}^{}\right)=\frac{1}{2}U^{T}U+\frac{g}{2}\sum_{n=1}^{N}b_{n}\zeta_{n}^{2}\\ s.t.z_{n}\left[U^{T}f\left(y_{n}\right)+q\right]=1-\zeta_{n},\;\left(n=1,\ldots ,N\right) \end{array}\right.$$

where U is the weight vector, *N* is the sample number, *g* is the penalty parameter, $b_{n}\left(n=1,\ldots ,N\right)$ are the weighting factors, *q* is the bias term, $\zeta_{n}^{}\left(n=1,\ldots ,N\right)$ are the error variables, and the output data are $z_{n}=\{\begin{array}{ll} 1, & y_{n}\;\in \;\mathrm{arc}\;\mathrm{faults}\\ 0, & y_{n}\in \mathrm{normal}\;\mathrm{states} \end{array}$.

To solve Equation (10), Lagrange multipliers $\beta_{n}^{}$ are used. The Lagrangian becomes

(11) $$L\left(U,\zeta_{n},\beta_{n},q\right)=W\left(U,\zeta_{n}^{}\right)-\sum_{n=1}^{N}\beta_{n}\left\{z_{n}\left[U^{T}f\left(y_{n}\right)+q\right]-1+\zeta_{n}\right\}$$

Based on the Karush–Kuhn–Tucker condition, the partial derivatives of U, $\zeta_{n}^{}$, $\beta_{n}^{}$, *q* in Equation (11) are equal to 0. One obtains

(12) $$\left\{ \begin{array}{l} U=\sum_{n=1}^{N}\beta_{n}f\left(y_{n}\right)\\ \beta_{n}=gb_{n}\zeta_{n}\\ z_{n}\left[U^{T}f\left(y_{n}\right)+q\right]-1+\zeta_{n}=0\\ \;\sum_{n=1}^{N}\beta_{n}z_{n}=0\;\left(n=1,\ldots ,N\right) \end{array}\right.$$

The solution can be given as

(13) $$\left(\begin{array}{cc} V+B_{g} & z\\ z^{T} & 0 \end{array}\right)\left(\begin{array}{c} \beta \\ q \end{array}\right)=\left(\begin{array}{c} I\\ 0 \end{array}\right)$$

where $V=f^{T}(x_{n})f(x_{p}),\;I={\left(1,\;\cdots ,\;1\right)}^{T},\;\beta ={\left(\beta_{1},\;\cdots ,\;\beta_{N}\right)}^{T},\;z={\left(z_{1},\;\cdots ,\;z_{N}\right)}^{T},\;n=1,\ldots ,N,\;p=1,\ldots ,N,$ and the diagonal matrix $B_{g}$ is

(14) $$B_{g}=\mathrm{diag}\left(\frac{1}{gb_{1}},\;\cdots ,\;\frac{1}{gb_{N}}\right)$$

According to the Mercer condition, the kernel function is $F(y_{n},y_{p})=f^{T}(y_{n})f(y_{p})$. Here, the radial basis function (RBF) is chosen as the kernel function

(15) $$F(y_{n},y_{p})=\exp\left(-{\left\|y_{n}-y_{p}\right\|}^{2}/\sigma^{2}\right),\;n=1,\ldots ,N,\;p=1,\ldots ,N$$

where σ^2^ is the RBF kernel parameter. The optimal parameters σ^2^ and *g* are obtained through the ten-fold cross-validation method [66]. The parameters β and *q* can be solved using the least square method [65].

Based on the above discussion, Equations (13) and (15) are used to solve Equation (10), and the identification results can be expressed as:

(16) $$z^{*}\left(y\right)=\mathrm{sgn}\left[\sum_{n=1}^{N}\beta_{n}z_{n}F\left(y,y_{n}\right)+q\right]$$

**Figure 18:**
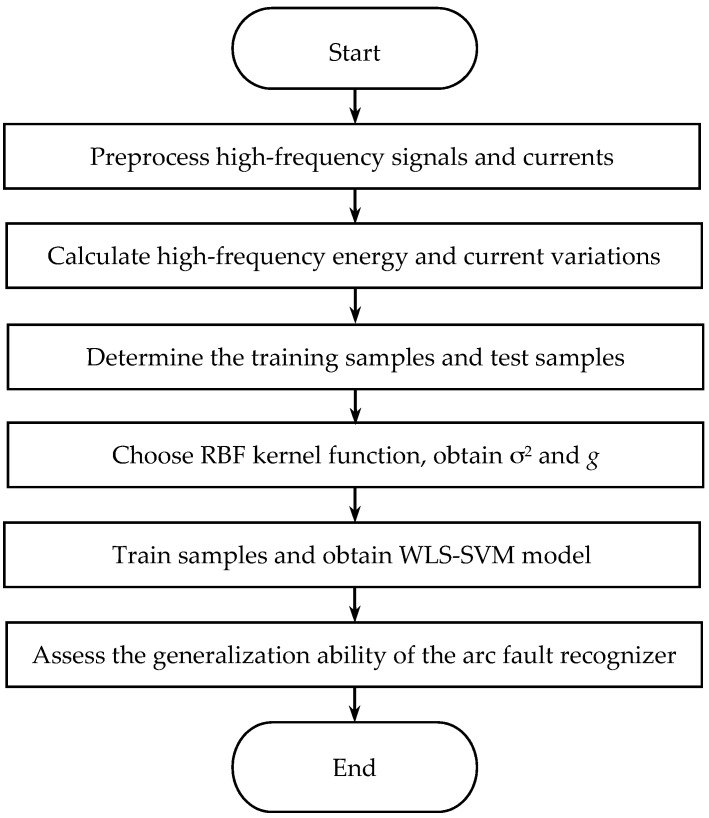
The flow chart of the arc fault detection algorithm.

Considering all the derivations and discussions above, a flow chart of the arc fault detection algorithm is designed, as shown in Figure 18. It will be implemented in the following.

The experimental data for nine typical loads under different states are selected as the sample set (***y***, ***z***). The input eigenvector ***y*** contains the current variation $y_{1}$ and the high-frequency energy $y_{2}$. The vector ***z*** represents the actual classification results, which were denoted by the authors. It is noted that “0” represents the normal state and “1” represents the arc fault state. The sample set includes 260 samples, as listed in Table 2, 200 of which are used as the training samples; the remaining samples are used as the test samples.

**Figure 19:**
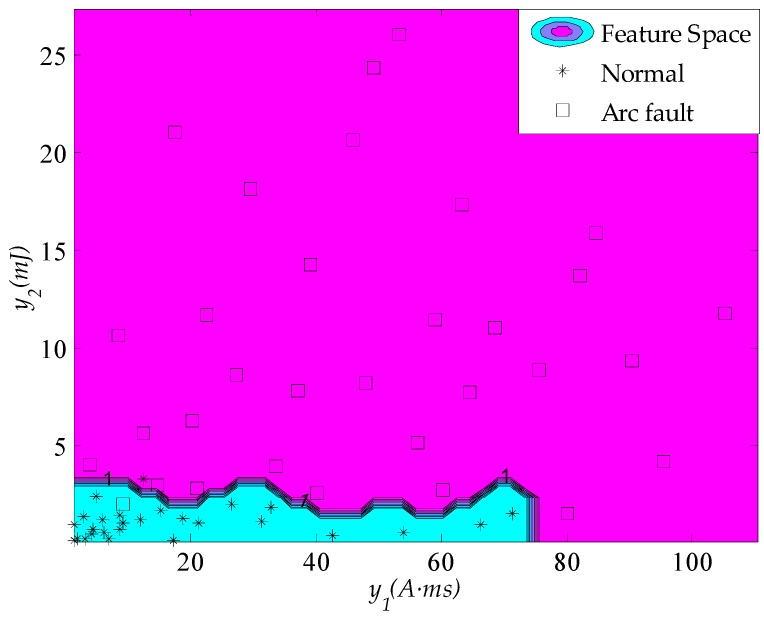
Identification results of load states.

In the arc fault detection algorithm, the arc fault recognizer is developed based on the training samples. The parameters σ^2^ = 0.095 and *g* = 3.26 are obtained via the ten-fold cross-validation method. The test samples are used to test the arc fault recognizer. The calculated classification results are compared with the actual classification results $z_{n}$. The generalization ability of the recognizer can be assessed through the error rate

(17) $$e_{M}=\frac{\sum_{n=1}^{M}\left|z_{n}^{*}-z_{n}\right|}{M}\times 100\%$$

where *M* is the test sample number and $z_{n}^{*}\left(n=1,\ldots ,M\right)$ are the calculative classification results. The identification results for the load states are shown in Figure 19. The symbol “□” represents an arc fault, and “*” represents the normal load state. The feature vector space is used to identify arc faults from the test samples. The optimal plane of classification between normal states and arc faults is found in the high-dimensional feature space. These identification results for load states are the globally optimal solution based on the obtained RBF kernel parameters. If the test sample appears in the arc fault feature space as shown in Figure 19, then an arc fault has occurred in the circuit; otherwise, there is no arc fault.

The confusion matrix of the arc fault detection algorithm after all the sample tests are completed is given in Table 3. The results show that the average error rate of the arc fault detection algorithm is 3.33%. Therefore, the arc fault identification rate is 96.67%. The developed algorithm demonstrates good generalization ability under different loads.

## 5. Designing AFD for the Early Detection of Electrical Fires

**Figure 20:**
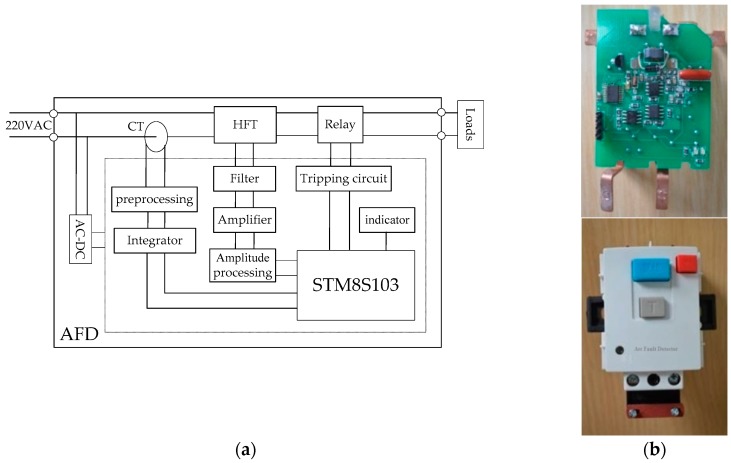
AFD: (**a**) schematic; (**b**) prototype.

The AFD is mainly composed of a transformer module, a signal conditioning module, a microprocessor module, an alarm module, and a power module. The AFD schematic is shown in Figure 20a. The transducer module contains an HFT and a CT, which were introduced in Section 2. These components are used to obtain the high-frequency signals and line currents, respectively, in low-voltage circuits. The obtained signals are filtered, amplified and integrated in the signal conditioning module. Then, the signals are sent to the microprocessor module for further processing. The arc fault detection algorithm is successfully applied in the STM8S103 module, which is selected as a microprocessor. The STM8S103 has 8 Kbytes of flash programmable memory, 640 bytes of true data Electrically Erasable Programmable Read-Only Memory (EEPROM) and 1 Kbyte of Random Access Memory (RAM); its maximum master clock frequency is 16 MHz. In the alarm module, the electrical relay is controlled by a silicon controlled rectifier. When arc faults occur, the microprocessor generates a tripping signal; then, the early alarm indicator lights up, and the relay interrupts the circuit power supply to prevent an electrical fire. A voltage of 220 V (50 Hz) is directly supplied to the power module. The AC-to-DC (direct current) convertor based on the LNK302 chip can output ±12 V and ±5 V for modules in the AFD. All modules are integrated into a complete AFD. An AFD prototype has been developed, as shown in Figure 20b. The prototype can be directly installed in a low-voltage circuit.

**Figure 21:**
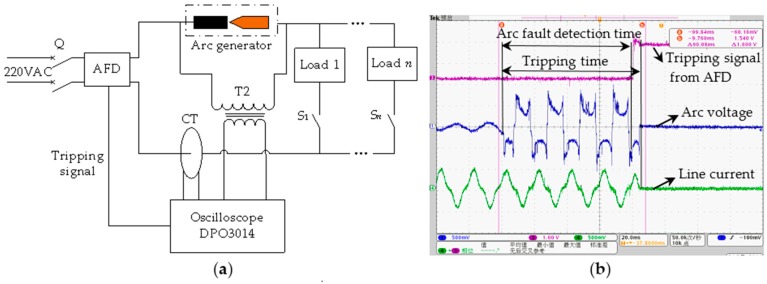
Tripping test: (**a**) schematic; (**b**) results.

According to various standards, including IEC 62606: 2013 and GB/T 31143-2014, numerous test experiments were performed to verify the detection effects under different loads. The schematic of the tripping test is shown in Figure 21a. The fault arc voltage, the line current and the tripping signal from the AFD were observed using a Tektronix DPO3014 oscilloscope, as shown in Figure 21b, and the tripping time of the AFD was 84 ms. The experimental schematic of cross talk is shown in Figure 10. When arc faults occurred in branch B1, AFD 1 could detect them in a timely manner and interrupt the circuit power supply before an electrical fire was produced to ensure the prevention of electrical fires. Moreover, AFD 2 did not alarm or trip. This is because the current variation was added as an important feature in the arc fault detection algorithm. The power frequency currents at 50 Hz are not easily influenced by cross talk. Therefore, the developed AFD can overcome the influences of cross talk and transient processes.

Test results under typical loads are shown in Table 4. The accuracy of the arc fault detection is very high, and the AFD exhibits good generalizability for use under different loads. Because some weak arc faults produced by electrical drills and computers were used in the tests, the features of arc faults were very similar to those of normal states. Hence, the AFD sometimes failed to detect these arc faults, as reflected under certain loads such as those of electrical drills and computers. This limitation will be improved in future work.

## 6. Conclusions

Arc faults occur in many electrical circuits and represent one of the most important causes of electrical fires. AFDs are urgently needed for the early detection of electrical fires. In this study, common arc fault features under different loads are extracted for the study of arc fault detection. The main conclusions are as follows:

1. High-frequency signals collected by an HFT noticeably increase when arc faults occur in circuits. However, they are strongly affected by cross talk.
2. The line currents of arc faults are often distorted or become unstable; however, current periodic integrals also clearly change under normal states, especially during transient processes.
3. High-frequency signal energy and current variations were extracted as the input eigenvector of the arc fault detection algorithm. An algorithm based on WLS-SVM was designed to identify arc faults from load states.
4. The arc fault detection algorithm was successfully applied in a STM8S103 microprocessor, and an AFD prototype was developed. The AFD was able to detect arc faults in a timely manner and interrupt the circuit power supply before electrical fires could be produced during testing. The prototype was not influenced by cross talk or transient processes. The detection accuracy was very high, and the AFD exhibited good generalizability under different loads.

Consequently, the AFD can be installed in low-voltage circuits to monitor circuit states in real-time to facilitate the early detection of electrical fires. The accuracy of the arc fault detection technique used by the prototype will be further enhanced in future research and development. Moreover, the AFD, the overcurrent protector, the ground fault circuit interrupter and other protection devices will be integrated to develop a combination circuit fault detection device. The combination device will be used to prevent all electrical fires.

## Figures and Tables

**Table 1:** Typical experimental loads.

Number	Load Name	Quantity	Unit Power (W)	Total Power (W)
1	Fluorescent lamp	2	40	80
2	Halogen lamp	6	50	300
3	Computer	1	350	350
4	Electrical drill	1	750	750
5	Dimming lamp	5	200	1000
6	Electric stove	1	1000	1000
7	Vacuum cleaner	1	1200	1200
8	Air conditioner	1	1500	1500
9	Air compressor	1	2200	2200

**Table 2:** The sample set of typical loads.

Sample Number	1	2	3	…	260
***y***_1_ (A·ms)	1.5513	22.1634	8.6517	…	40.6125
***y***_2_ (mJ)	0.9726	11.7258	1.4209	…	14.2903
***z***	0	1	0	…	1

**Table 3:** Confusion matrix for arc fault detection algorithm.

Actual Classifications	Calculative Classifications	
	Normal	Arc Fault
Normal	27	1
Arc Fault	1	31

**Table 4:** Test results under typical loads.

Number	Load Name	Start or Stop Condition	Arc Fault Detection Accuracy
1	Computer	without tripping	98%
2	Halogen lamp	without tripping	100%
3	Electrical drill	without tripping	96%
4	Dimming lamp	without tripping	100%
5	Vacuum cleaner	without tripping	100%
6	Air conditioner	without tripping	100%
7	Air compressor	without tripping	100%
8	Fluorescent lamp and electric stove	without tripping	100%
